# Brain-targeting delivery of MMB4 DMS using carrier-free nanomedicine CRT-MMB4@MDZ

**DOI:** 10.1080/10717544.2021.1968977

**Published:** 2021-09-13

**Authors:** Yimeng Du, Jing Gao, Hui Zhang, Xiaohui Meng, Dong Qiu, Xiang Gao, Aiping Zheng

**Affiliations:** a State Key Laboratory of Toxicology and Medical Countermeasures, Beijing Institute of Pharmacology and Toxicology, Beijing, China; b Beijing National Laboratory for Molecular Sciences, CAS Research/Education Center for Excellence in Molecular Sciences, Institute of Chemistry, Chinese Academy of Sciences, Beijing, China

**Keywords:** MMB4 DMS, nanocrystal, molecular simulation, core–shell nanoparticle, protein corona

## Abstract

Brain-targeting delivery of 1,1′-methylenebis[4-[(hydroxyimino)methyl]-pyridinium] dimethanesulfonate (MMB4 DMS) is limited by its hydrophilic property and chemical instability. In order to solve this problem, herein, we develop a facile protocol through combining antisolvent precipitation and emulsion-solvent evaporation method to synthesize midazolam (MDZ) coated MMB4 DMS (MMB4@MDZ) nanoparticles. The as-prepared MMB4@MDZ had a MMB4 DMS nanocrystal (MMB4-NC) core and a MDZ shell. The MDZ shell prevented the MMB4-NC core from contacting the aqueous environment, and thus, guaranteed the chemical stability of MMB4 DMS. Most charmingly, the iron mimic cyclic peptide CRTIGPSVC (CRT) was modified on MMB4@MDZ surfaces to produce CRT-MMB4@MDZ which was endowed with ability to absorb transferrin (Tf)-abundant corona. Taking advantages of the Tf-abundant corona, CRT-MMB4@MDZ achieved transferrin receptor (TfR)-mediated brain-targeting delivery. With the fascinating chemical stability and brain-targeting delivery effect, CRT-MMB4@MDZ showed great clinical transform prospect as a brand-new nanomedicine. Of particular importance, this work promised not only a core–shell carrier-free nanomedicine platform for effective delivery of unstable water-soluble drug, but also a protein corona-manipulating strategy for targeting delivery.

## Introduction

Organophosphorus compounds (OPCs) have been widely used as pesticides and developed into chemical warfare nerve agents (CWNAs). Every year, hundreds of thousands of intoxications by OP pesticide occurred due to suicides or accidents worldwide (Mew et al., 2017). Moreover, CWNA has been repeated disseminated in military conflicts, in assassination attempts (assassination against Kim Jong-Nam in Malaysia), and in terrorist scenarios (the Tokyo subway attack) (John et al., 2018). The OPC are highly toxic, and exposure to either OPC class can be a fatal threat to civilians or military personnel (Okumura et al., 2005; Baker, 2013; Dolgin, 2013). The hydrophobic OPC can easily spread across physical barriers to irreversibly inhibit the enzyme acetylcholinesterase (AChE) (Marrs, 1993), producing continuous stimulation of cholinergic fibers throughout the peripheral and central nervous systems (CNSs). The necessity of AChE reactivation in CNS has long been reported according to the fact that survivors without reactivation of AChE in CNS suffered long lasting brain damage (Voicu et al., 2015).

The causal treatment of OPC poisoning is administration of reactivators to reactivate the AChE inhibited by OPC. Enormous research efforts have been devoted to develop reactivator capable of crossing the blood–brain barrier (BBB) to rescue the OP inhibited AChE in CNS (Worek & Thiermann, 2011; Chambers et al., 2020). Currently, 1,1′-methylenebis[4-[(hydroxyimino)methyl]-pyridinium] dimethanesulfonate (MMB4 DMS) is proposed as a leading oxime reactivator exhibiting the best overall efficacy against a wide spectrum of OPC (Wilhelm et al., 2014). Nevertheless, its hydrophilic property limited the penetration of MMB4 DMS across BBB, and the chemical instability of MMB4 DMS in aqueous environment is a critical issue for its clinical application. Research efforts should be devoted to develop a novel drug delivery strategy for MMB4 DMS to improve its stability in blood circulation and enhance its brain-targeting delivery.

The U.S. FDA-approved therapeutic regimen to treat OP-induced toxicity entails administration of AChE oxime reactivator, an antimuscarinic agent and an anticonvulsant for both symptomatic and causal treatments. Specifically, due to the reason that CNS exposure to OP compounds can induce convulsive seizures and status epilepticus (SE) and thus cause profound brain damage (Chen, 2012; Hobson et al., 2018; Scholl et al., 2018), the application of anticonvulsant was necessary for the treatment of CNS. In recent decade, midazolam (MDZ) has been reported to protect against OP-induced brain damage in rats as a more effective benzodiazepine anticonvulsant (RamaRao et al., 2014; Chapman et al., 2015; Reddy & Reddy, 2015). FDA and French army are currently considering approval of MDZ as treatment of OP compounds induced seizures (McDonough, 2002; Wu et al., 2018). Inspired by the hydrophobic property of MDZ and the synergistic application of MMB4 DMS and MDZ to CNS, herein we proposed a core–shell carrier-free nanomedicine consisting of a MMB4 DMS core and a MDZ shell. The MDZ shell could confer protection to the MMB4 DMS core in aqueous environment to improve the chemical stability of MMB4 DMS.

MDZ is hydrophobic at physiology pH, but become more hydrophilic in an acidic environment via the opening of the imidazole ring (Andersin, 1991). In view of this, transferrin receptor (TfR) mediated brain targeting delivery received our research attentions. It is well known that the iron containing transferrin (Tf) in blood circulation can bind with the TfR on BBB. Following the TfR-mediated endocytosis, pH within the early endosome reduced to about 5.5 leading to the release of iron from the Tf/TfR complex (Van Renswoude et al., 1982; Bali et al., 1991). Taking advantages of the acidic environment, in this study, the TfR-mediated BBB crossing strategy is proposed for MDZ coated MMB4 DMS (MMB4@MDZ) nanoparticles to accelerate the MDZ releasing at the beginning of its journey into brain. The cyclic peptide CRTIGPSVC (CRT) is an iron-mimicking peptide which can non-disruptively bind with Tf (Liu et al., 2018). Therefore, a CRT-modified nanoparticle can specifically bind with endogenous Tf, and thus, can achieve TfR-mediated translocation across BBB. In recent years, several studies have reported that the conjugation of peptide CRT to drug nanocarriers yields TfR-mediated drug delivery (Kang et al., 2015; Zhang et al., 2016; Falanga, 2018; Liu et al., 2018; Zhang et al., 2020). However, to the best of our knowledge, its potential as a ligand for modification of carrier-free nanomedicine has scarcely been investigated.

In this study, core–shell MMB4@MDZ nanoparticles were facilely synthesized via combination of antisolvent precipitation and emulsion technology. The as-synthesized MMB4@MDZ tremendously improved the chemical stability of MMB4 DMS in aqueous environment. Furthermore, surface modification of MMB4@MDZ with CRT realized the specific absorption of Tf. The Tf corona-transformed nanomedicine CRT-MMB4@MDZ could be delivered across BBB through TfR mediated route. This study paved a way to expand the core–shell nanoparticle strategy for delivery of unstable water-soluble drug, and also to exploit the Tf corona targeting strategy for pH-sensitive drug carriers.

## Materials and methods

### Materials

All the reagents were used as received. Midazolam was purchased from Astellas Pharma Inc. (Tokyo, Japan). MMB4 DMS was supplied by Beijing Institute of Pharmacology and Toxicology (Beijing, China). The cyclic peptide CRT (sequence: CRTIGPSVC) was chemically synthesized by the Chinese Peptide Co., Ltd. (Hangzhou, China). The ChromPure mouse Tf was purchased from Jackson ImmunoResearch Laboratories (West Grove, PA). Methanol, isoamyl acetate (IA), trifluoroacetic acid (TFA), tetraethylammonium chloride (TEAC), and sodium dodecyl sulfate (SDS) were purchased from Sigma-Aldrich (St. Louis, MO). Acetic acid was purchased from Sinopharm Chemical Reagent Co., Ltd. (Shanghai, China). MTT kits were purchased from Immutopics Ltd. (San Clemente, CA). Dulbecco's modified Eagle's medium (DMEM), fetal bovine serum (FBS), and penicillin/streptomycin were purchased from Gibco (Dublin, Ireland). Other reagents, if not specified, were obtained from Chinese Medicine Group.

### Computational simulations

Molecular structures of IA, MDZ, and SDS were built using Materials Studio 2017. Dynamic molecular simulation technique was adopted to probe the interfacial behavior of MDZ molecules in the O/W emulsion system. In order to investigate the formation process of the MDZ-NP, two simulation systems were constructed. The firs system (named ‘S-I’) consisted of a MDZ IA solution in contact with a SDS water solution. Briefly, a simulation box of 6×6×8 nm^3^ was initially filled with water molecules, and then, the box was expanded in the *z* direction to a length of 16 nm and IA molecules were introduced to fill the empty space. Subsequently, solutions were built by randomly replacing solute (MDZ and SDS) molecules with solvent (water and IA) molecules in the simulation box. As a result, S-I was setup to simulate the formation of the O/W emulsion. After 100 ns simulation of S-I, the second system (named ‘S-II’) was further constructed by randomly halving the IA molecules. The details of the simulation systems are given in Table 1.

**Table 1.** Model details for the two simulation systems.

Simulation system	No. of water molecules	No. of SDS molecules	No. of IA molecules	No. of MDZ molecules	Box dimension (nm) *x*, *y*, and *z*
S-I	4000	50	1000	75	6, 6, 16
S-II	4000	50	500	75	6, 6, 10

Molecular dynamics (MD) simulation was carried out utilizing the GROMACS package (version 4.5.5) with periodic boundary conditions applied in all three directions. First, a 5000-step energy minimization was performed to the simulation system, followed by pre-equilibrations in the NVT ensemble (constant number of particles, volume, and temperature). A constant temperature (298 K) and a constant pressure (1 atm) were maintained by the Berendsen and Parrinello-Rahman coupling scheme. All the bonds were constrained by the LINCS algorithm, and the equations of motion were integrated with a step of 2 fs. The particle mesh Ewald (PME) method was applied to handle long-range electrostatic interactions. A twin-range cutoff scheme was used for short-range electrostatics and van der Waals (vdW) interactions with a cutoff value of 1.0 nm. Molecular dynamics production runs were performed for 100 ns, and trajectories were stored every 10 ps.

### Preparation of MMB4@MDZ nanoparticles

MMB4 DMS nanocrystals (MMB4-NCs) were prepared by an antisolvent precipitation method. We have previously evaluated solvents and antisolvents by investigating the size distribution of precipitated MMB4-NCs (unpublished). Methanol and IA were finally chosen to be solvent and antisolvent, respectively, and high quality MMB4-NCs could be successfully synthesized without addition of stabilizer. Generally, MMB4 DMS were dissolved in methanol to produced MMB4 DMS solution, and then 1.5 mL of MMB4 DMS solution was injected into 30 mL IA under vigorous stirring (8000 rpm). Immediately, the solute MMB4 DMS was precipitated and a MMB4-NC suspension formed. MMB4 DMS solution with different concentrations (0.4, 0.2, 0.1 and 0.05 mg/mL) were studied for the synthesis of MMB4-NCs.

To construct the core–shell MDZ coated MMB4 DMS (MMB4@MDZ) nanoparticles, an oil-in-water (O/W) emulsion protocol was performed by incorporating MMB4-NCs and dissolved MDZ into the oil phase of emulsion. Briefly, 3 mg MDZ was dissolved into 30 mL of the MMB4-NC IA suspension. The O/W emulsion was generated by mixing 30 mL of MMB4-NC/MDZ suspension with 450 mL of 0.6 mg/mL SDS solution (in MilliQ water) through vigorous stirring (600 rpm). IA was allowed to be evaporated at room temperature under continuous stirring. After IA was completely removed, the growth of the MDZ shells was accomplished and the MMB4@MDZ nanoparticles were collected by ultrafiltration centrifugation. The as-synthesized MMB4@MDZ nanoparticles were then washed with MilliQ water for five times to remove SDS. MDZ nanoparticles (MDZ-NPs) without MMB4-NC cores were also synthesized through this O/W emulsion protocol by only employing 0.1 mg/mL MDZ IA solution as the oil phase.

### Preparation of CRT-MMB4@MDZ, Tf-MMB4@MDZ, and BSA-MMB4@MDZ nanoparticles

CRT-MMB4@MDZ nanoparticles were prepared by associating CRT on the surface of MMB4@MDZ nanoparticles, respectively. Briefly, excess CRT was added into MMB4@MDZ nanoparticle suspension, which was then mixed by mild stirring for 2 h at room temperature. Unbound CRT was removed by repeated ultrafiltration centrifugation. The modification density of CRT was quantified using the BCA protein assay. Briefly, the prepared CRT-MMB4@MDZ was pelleted by centrifugation and resuspended in PBS, and the CRT concentration in this CRT-MMB4@MDZ suspension was measured by using a commercial BCA protein assay kit (Solarbio Life Sciences, Beijing, China). Subsequently, 50 μL of the CRT-MMB4@MDZ suspension was dissolved by 200 μL methanol. After centrifugation (15,300 rpm, 30 min), the MMB4 DMS concentration was measured by high-performance liquid chromatography (HPLC). Finally, the modification density was calculated as mg of CRT per mg of MMB4 DMS.

Besides CRT-MMB4@MDZ, Tf-modified MMB4@MDZ (Tf-MMB4@MDZ) nanoparticles and BSA-modified MMB4@MDZ (BSA-MMB4@MDZ) nanoparticles were also prepared by incubating MMB4@MDZ in Tf solution or BSA solution, respectively. The modification density of Tf was also determined with the method described above.

### Characterization

The mean particle size, the polydispersity index (PDI), and the zeta potential of nanoparticles were measured by dynamic light scattering (DLS) (Malvern Zetasizer Nano ZS, Malvern Instrument, Malvern, UK). All measurements were carried out in triplicate and performed at 25 °C. Nanoparticle morphology was observed under transmission electron microscopy (TEM) (JEOL-2100F electron microscope, Akishima, Japan). The X-ray diffraction patterns of the unprocessed MMB4 DMS, the unprocessed MDZ, MMB4-NCs, and MDZ-NPs were obtained by using a Bruker aXS D8 X-ray diffractometer (Bruker, Billerica, MA). Each sample was scanned over an angular range 2*θ* from 3° to 40° with a scan speed of 0.1 s per step.

### Determination of MMB4 DMS concentration and MDZ concentration by HPLC

The concentration of MMB4 DMS and MDZ was measured by HPLC. HPLC analyses were conducted by using Agilent 1200 HPLC equipped with an UV absorbance detector (Santa Clara, CA), an autosampler and in-line degasser. Separation was achieved using a C_18_ column (Agilent C18, Santa Clara, CA, 5 μm, 4.6 mm × 250 mm) at a flow rate of 1 mL/min and an injection volume of 10 μL. Mobile phase A: 0.17 mg/mL TEAC in MilliQ water with pH adjusted to be 3.0 by adding phosphoric acid, 45%. Mobile phase B: acetonitrile, 55%. The detection wavelength was 290 nm for MMB4 DMS and 220 nm for MDZ.

### Stability study

The chemical stability of MMB4 DMS in 50 μg/mL MMB4 DMS solution (in MilliQ water) and MMB4@MDZ suspension (at a concentration of 50 μg/mL MMB4 DMS-equiv.) were compared. Aliquot of samples was sealed in glass injection vials, and then, were placed in 60 °C incubator. At predetermined time points, the samples were analyzed by HPLC to measure the content of MMB4 DMS. The MMB4-NC suspension in IA (at a concentration of 50 μg/mL MMB4 DMS-equiv.) was investigated by incubated under 25, 40 and 60 °C for 12 h. After incubation, the content of MMB4 DMS was measured and normalized relative to the un-treated MMB4-NC. In particular, for the HPLC analysis, the MMB4-NCs and MMB4@MDZ nanoparticles were dissolved in methanol.

### *In vitro* drug release

The release profile was measured under the neutral condition mimicked physiological condition using phosphate buffer saline (PBS, pH 7.4), and under an acidic condition mimicked the acidic environment during TfR-mediated delivery using PBS adjusted to pH 5.5 with acetic acid. For evaluating MDZ release behavior, unprocessed MDZ powder and MDZ-NP were suspended in 2 mL releasing buffer and transferred into dialysis membrane cassettes (Slide-A-Lyzer G2, Thermo Scientific, Rockford, IL) with a molecular weight cutoff of 10 kDa. The cassettes were then immersed in 100 mL of releasing buffer under magnetic stirring (200 rpm) at 37 °C. At predetermined time points (1, 2, 3, 4, 5 and 6 h), 2 mL of samples were withdrawn from the outside releasing buffer for HPLC analysis and 2 mL of fresh releasing buffer were supplemented. The MDZ and MMB4 DMS concentrations were analyzed by HPLC as described above.

### Evaluation of the specific binding of CRT-MMB4@MDZ with Tf

BSA-MMB4@MDZ was prepared as a control sample. The stock solutions of CRT-MMB4@MDZ and BSA-MMB4@MDZ were prepared at a concentration of 1000 μg/mL MMB4 DMS-equiv. in PBS. Subsequently, the stock solutions were serially diluted with PBS to generate standard solutions with MMB4 DMS concentration of 15,000, 7500, 1500, 750, 150 and 0 ng/mL. All standard solutions were freshly prepared before the experiment. One hundred micrograms per milliliter Tf solution (in PBS) was added into a 96-well plate to incubate at 4 °C overnight. After incubation, the wells were washed by PBS for five times. Subsequently, the CRT-MMB4@MDZ and BSA-MMB4@MDZ standard solutions were added into the 96-well plate with a volume of 100 μL per well. After 1 h incubation at 37 °C, the plate was washed by PBS for five times. Finally, methanol was added into the plate with a volume of 200 μL per well to dissolve the bound nanoparticles. MMB4 DMS concentration of the resultant methanol solutions was measured by HPLC.

### Cytotoxicity analysis

Mouse brain endothelial (bEnd.3) cells were purchased from the American Type Culture Collection (ATCC, Manassas, VA). The cells were cultured in culture median (DMEM supplemented with 10% FBS and 1% penicillin/streptomycin solution) and maintained in 5% CO_2_/95% humidified air at 37 °C. The viability of bEnd3 cells treated with MMB4 DMS solutions (in culture median), MMB4@MDZ, Tf-MMB4@MDZ, and CRT-MMB4@MDZ was measured by MTT assay. Briefly, cells were seeded in a 96-well plate at a density of 1 × 10^4^ cells/well and cultured for 24 h before the experiment. The MMB4 DMS solutions with different concentrations (10, 1, 0.1  and 0.01 mg/mL), MMB4@MDZ, Tf-MMB4@MDZ, and CRT-MMB4@MDZ suspensions with the same concentration (1 mg/mL MMB4 DMS-equiv.) were applied to corresponding wells (*n* = 8). Culture median was used as a negative control and 10% DMSO as a positive control. Following 6 h incubation, the test solutions were removed, cells were washed three times, and cell viability was quantified by a commercial MTT kit. The MTT reagent in culture medium was added for a further 3 h incubation at 37 °C. After aspirating the medium of each well, 200 μL of formazan was added to each well to dissolve the formazan crystal. The resulting absorbance was read at 490 nm using Varioskan LUX multimode microplate reader. The relative cell viability was normalized relative to the negative control cells.

### Evaluation of transepithelial transport

The bEnd.3 cells were seeded onto polycarbonate 24-well transwell apparatus (Falcon Cell Culture Insert) at a density of 10 × 10^4^ cells/well. The culture medium was changed every two days. The integrity was monitored using an epithelial voltohmmeter (Millicell ERS) to measure the transepithelial electrical resistance (TEER) and only the *in vitro* BBB model with TEER above 200 Ω cm^2^ could be used for the following experiments. MMB4 DMS was dissolved in culture medium to prepare 25 μg/mL solution. The nanoparticle samples (MMB4@MDZ, Tf-MMB4@MDZ, and CRT-MMB4@MDZ) were dispersed in culture medium and Tf was added to prepare MMB4@MDZ, Tf-MMB4@MDZ, and CRT-MMB4@MDZ suspensions (25 μg/mL MMB4 DMS-equiv.) supplemented with 1 mg/mL free Tf. The freshly prepared solution and suspensions were added into different apical cell culture inserts (donor chamber) in a volume of 0.2 mL. At predetermined time points (15, 30, 45, 60, 90, 120, 180, 240, 300 and 360 min), 100 μL of medium was collected from basolateral chambers (receptor chamber) and equal volume of fresh and pre-warmed media was supplemented. The collected medium samples were mixed with 400 μL methanol to precipitate proteins. After vortex (1 min) and centrifugation (15,300 rpm, 30 min), the supernatant was obtained and analyzed by HPLC to measure the concentration of MMB4 DMS. Six replicates of each experiment were performed. The TEER value of each insert was monitored during experiment to explore the integrity of cell monolayer.

### Transcytosis study

Seven days before experiment, bEnd.3 cells were cultured in confocal dishes. In order to track the transcytosis route of nanoparticles, Cy7@MDZ was synthesized by replacing MMB4 DMS with sulfo-cyanine7 carboxylic acid (Cy7, Dalian Meilun Biotechnology Co., Ltd., Dalian, China). Subsequently, the CRT-Cy7@MDZ was prepared by the same preparation method for CRT-MMB4@MDZ as described above. To assess the mechanism of cellular uptake, the internalization of CRT-Cy7@MDZ was visualized by confocal laser scanning microscopy (CLSM) (LSM-880, Carl Zeiss, Oberkochen, Germany) after co-incubating it with Tf (Jackson Immunoresearch Labs, West Grove, PA) and a phycoerythrin-labeled rat anti-mouse TfR monoclonal antibody (R17217; Abcam, Cambridge, UK) (TfR-Ab) for 4 h. In addition, the intracellular localization of Cy7@MDZ was also visualized by CLSM after co-incubating with Lyso-Tracker Red (Beyotime Biotechnology, Shanghai, China) and Golgi-Tracker (Beyotime Biotechnology, Shanghai, China). Finally, the cell nuclei were stained by Hoechst 33342 (Beyotime Biotechnology, Shanghai, China). The captured CLSM images were then analyzed using the ImageJ software (NIH, Bethesda, MD) to calculate the overlap coefficient.

### *In vivo* imaging

The animal experiments were approved by the Animal Ethic Committee at Beijing Institute of Pharmacology and Toxicology (ethics code permit no. SCXK-(Beijing) 2007-004 and SCXK-(Beijing) 2007-003). The approval has been received before the beginning of the animal researches. All the animal experiments were carried out following the National Institutes of Health guide for the care and use of Laboratory animals (NIH Publications No. 8023, revised 1978). Healthy male C57 mice (22–24 g) were purchased from the Beijing Weitonglihua Experimental Animal Technology Co., Ltd. (Beijing, China). The mice were housed in standard cages, maintained in a breeding room with a temperature of 25 ± 1 °C, and given free access to food and water. The mice were intravenously (i.v.) administered with free Cy7 (Cy7 saline solution), Cy7@MDZ suspension (in saline), and CRT-Cy7@MDZ suspension (in saline) at a dose of 25 mg/kg Cy7. After administration, the mice were imaged with a Lumina LT device (Perkin-Elmer, Waltham, MA) at scheduled time intervals (0, 1, 2, 4 and 6 h). Throughout the imaging experiments, the mice were kept under anesthesia (3% isoflurane flow). At the end of the experiment (6 h post-injection), the region-of-interest (ROI) analysis was carried out to measure the intensity of the fluorescence in the brain region for the Cy7@MDZ and the CRT-Cy7@MDZ group.

### Pharmacokinetic study

Healthy male SD rats (180 ± 10 g) were purchased from the Beijing Weitonglihua Experimental Animal Technology Co., Ltd. (Beijing, China). The rats were randomly divided into three groups and i.v. injected with MMB4 DMS saline solution, MMB4@MDZ suspension (in saline) and CRT-MMB4@MDZ suspension (in saline) at a single dose of 3 mg/kg (*n* = 3). Whole blood samples (500 μL) were withdrawn from the retro-orbital sinus and collected into heparinized tubes at 0, 5, 10, 15, 30, 60, 90, 120 and 180 min after injection. The blood samples were then centrifugated at 5000 rpm for 20 min and the supernatant plasma was collected for HPLC analysis. Generally, 200 µL of plasma was treated with 800 µL of methanol to precipitate the proteins. The mixture was vortexed for 1 min and centrifugated at 15,300 rpm for 30 min, and the obtained supernatant was analyzed by HPLC.

### Biodistribution study

To assess the tissue distribution of MMB4 DMS, the SD rats (male, 180 ± 10 g) were i.v. injected via tail vein with MMB4 DMS solution, MMB4@MDZ and CRT-MMB4@MDZ at a dose of 5 mg/kg (*n* = 3). Rats were sacrificed at 0 min or 4 h after drug administration to collect cerebrospinal fluid (CSF). In addition, the liver, spleen, lung, kidney and brain were collected at 4 h after drug administration. The tissue samples were homogenized, and the tissue homogenate and CSF were analyzed with the same procedure. Briefly, 100 μL of tissue homogenate or CSF was mixed with 400 μL of methanol. After 1 min vortex and centrifugation (15,300 rpm, 30 min), the supernatant was collected for HPLC analysis.

### Analysis of protein corona

MMB4@MDZ and CRT-MMB4@MDZ nanoparticles were incubated with rat plasma (Weitong Lihua, Beijing, China) at 37 °C for 2 h. After incubation, the protein corona-nanoparticle (PC-NP) complexes were separated by centrifugation at 15,300 rpm (4 °C) for 8 h. The pellet was washed with PBS for three times to remove the proteins with low affinity (i.e. the soft protein corona). Qualitative analysis of the protein corona samples was first carried out using sodium dodecyl sulfate polyacrylamide gel electrophoresis (SDS-PAGE). The PC-NP samples were lysed with lysis buffer (8 M urea, 100 mM TEAB, pH 8.5), and the lysate was centrifuged at 12,000×*g* for 15 min at 4 °C. Subsequently, the supernatant was further treated with 10 mM DL-dithiothreitol (DTT) for 1 h at 56 °C, and alkylated with sufficient iodoacetamide (IAM) for 1 h at room temperature in the dark. The protein sample was then loaded to 12% SDS-PAGE gel electrophoresis, wherein the concentrated gel was run for 20 min at 80 V and the separation gel was run for 90 min at 120 V. Finally, the gel was stained by coomassie brilliant blue R-250 and decolored until the bands were visualized clearly.

For LC-MS/MS analysis, proteins in the PC-NP sample were digested by trypsin at 37 °C for 4 h, and then digested with trypsin and CaCl_2_ overnight. After digestion, the sample was loaded to C18 desalting column, washed with washing buffer (0.1% formic acid, 3% acetonitrile) three times, then eluted by elution buffer (0.1% formic acid, 70% acetonitrile). The eluents of each sample were collected and lyophilized. For LC-MS/MS analysis, solution A (100% water, 0.1% formic acid) and B solution (80% acetonitrile, 0.1% formic acid) were prepared. The above-mentioned lyophilized sample was dissolved in solution A and centrifugated at 14,000×*g* for 20 min at 4 °C. After centrifugation, the supernatant was analyzed by LC-MS/MS using an ultimate 3000 nanoLC system (Dionex Inc., Sunnyvale, CA) and a tandem mass spectrometry (MS/MS) in a Q Exactive (Thermo Scientific Inc., Waltham, MA). Briefly, the peptides were separated in an analytical column (15 cm × 150 μm, 1.9 μm), and the separated peptides were analyzed by Q Exactive^TM^ HF-X mass spectrometer (Thermo Fisher, Waltham, MA), with ion source of Nanospray Flex™ (ESI), spray voltage of 2.1 kV, and ion transport capillary temperature of 320 °C. Data dependent analysis was performed. Full scan range was from *m/z* 350 to 1500 with resolution of 60,000 (at *m/z* 200), an automatic gain control (AGC) target value was 3 × 10^6^ and a maximum ion injection time was 20 ms. The top 40 precursors of the highest abundant in the full scan were selected and fragmented by higher energy collisional dissociation (HCD) and analyzed in MS/MS, where resolution was 15,000 (at *m/z* 200), the AGC target value was 1 × 10^5^, the maximum ion injection time was 45 ms, a normalized collision energy was set as 27%, an intensity threshold was 2.2 × 10^4^, and the dynamic exclusion parameter was 20 s. The all resulting spectra were searched by the search engines via Proteome Discoverer 2.2 (PD 2.2, Thermo, Waltham, MA). The retrieval results were further filtered by PD 2.2 to validate the analysis results. Peptide spectrum matches (PSMs) with a credibility of more than 99% was identified PSMs. The identified protein should contain at least one identified peptide. The identified PSMs and protein were verified by FDR and only the data with FDR smaller than 1.0% were retained.

### Statistical analysis

Data are presented as mean ± standard deviation (SD). Statistical analysis was performed using one-way analysis of variance (ANOVA) to determine the significance among groups. A value of *p*<0.05 was considered to indicate statistical significance.

## Results and discussion

### Computational simulations

**Figure 1.**
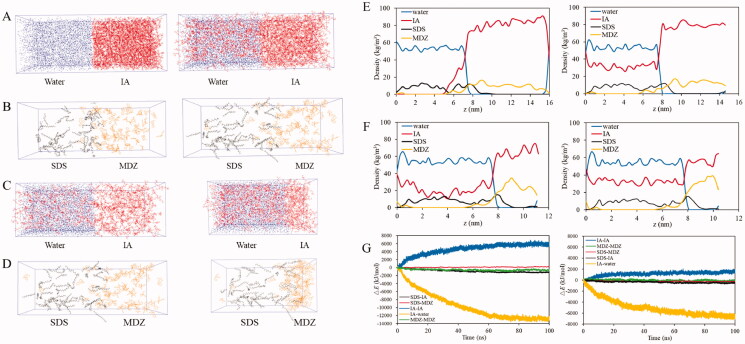
Initial (*t* = 0 ns, left panel) and final (*t* = 100 ns, right panel) configurations of the simulation system (A, B) S-I and (C, D) S-II. One simulation box is shown in two configurations for clarity. Water and IA are simultaneously shown in blue and red, respectively. SDS and MDZ are simultaneously shown in black and orange, respectively. Initial (*t* = 0 ns, left panel) and final (*t* = 100 ns, right panel) mass density distribution in the *z* direction for each individual component in the simulation system (E) S-I and (F) S-II. (G) Time evolution of change in interaction energy (Δ*E*) between individual components in IA-IA, SDS-MDZ, MDZ-MDZ, SDS-water and IA-water.

Taking advantages of the hydrophobic property of MDZ, MDZ-NPs can be formed through an emulsion-solvent evaporation method. MDZ was dissolved in a volatile water-immiscible solvent, IA, and then was dispersed in water with the presence of SDS to form an O/W emulsion system. After removal of the volatile IA MDZ-NPs were obtained, but the synthesis progress was difficult to be thoroughly studied using experiments. As a result, the powerful MD simulation technology was employed to simulate the molecular behaviors near the O/W interfaces. As shown in Figure 1(A), the initial configuration (*t* = 0 ns) of S-I consists of water molecules and IA molecules distributing separately. In the following simulation, the IA molecules constantly diffused into the water phase, while the MDZ molecules basically concentrated in the IA phase. This is in agreement with the experimental phenomenon. In order to quantitatively investigate the molecular movement, the mass density distribution of each type of molecule along the *z* direction (perpendicular to the O/W interface) is plotted. As shown in Figure 1(E,F), the left panels show the mass density distribution profiles at *t* = 0 ns, which is just after the pre-equilibrations, while the right panels show the profiles obtained after 100 ns simulation. The initial mass density distribution profile of S-I shows that water and IA separately occupy half of the simulation box. The four density profiles of water have minor differences. On the other hand, for both S-I and S-II, the IA profiles illustrate that the density of IA in the water phase increased at the end of simulation. In S-I, the mass density profile of SDS and MDZ both uniformly distributes in the water phase and the IA phase, respectively, and partly overlaps near the interface. In the second simulation system S-II, the density profiles of SDS and MDZ change from uniform to having a peak. The locations of the O/W interface and the density profile peaks are listed in S-II (Table 2). The position of the O/W interface was defined using the intersect of the density profile for water and IA. The MDZ molecules condensed in the IA phase and form a peak in its density profile at 9.04 nm and 9.55 nm before and after simulation. The amphiphilic property of SDS molecules drove them to move from bulk water to the interface and even to condense in the IA side, evidenced by a peak in its density profile appearing at 7.91 nm and 8.07 nm before and after simulation. Interestingly, SDS peaks are located closer to the interface than the MDZ peaks, suggesting the SDS molecules tended to be not packed into the MDZ-NPs.

**Table 2.** The *z*-coordinates (*z*, nm) of the O/W interface and peaks in the mass density profiles of SDS and MDZ in the simulation system S-II.

Simulation time (ns)	Interface	SDS			MDZ		
	*z*	*z*	*ρ*	*d*	*z*	*ρ*	*d*
0	7.57	7.91	15.30	0.34	9.04	34.89	1.47
100	7.72	8.07	15.04	0.35	9.55	38.20	1.83

The mass density (*ρ*, kg/m^3^) at the peaks, and the distance (*d*, nm) between the peaks and the interface were also calculated.

In order to investigate the driving forces for the formation of the hollow MDZ-NP, energetics analyses were performed. The changes in interaction energy (Δ*E*) between individual components are plotted as functions of time in Figure 1(G), and all the Δ*E* values are collected at the end of simulation (Table 3). In both S-I and S-II, Δ*E* between IA and water significantly decreases with time, which indicates that the attraction between the IA and water drove IA molecules to constantly disperse into the bulk water. The Δ*E* between IA molecules increases with time, implying an energy penalty as IA molecules move away from the bulk IA. In addition, the reduction of the interaction energy between MDZ molecules in S-I and S-II demonstrated the tendency of MDZ precipitation with the diffusion of IA. The reduction in the interaction energy between SDS and IA provides the driving force for the stabilization of SDS molecules on the interface, and it is the vdW interactions made major positive contribution as revealed by the large negative value of Δ*E*_vdW_. Meanwhile, in S-I, the interaction between SDS and MDZ (Δ*E*_elec_ = 43.83 kJ/mol, Δ*E*_vdW_ = 178.93 kJ/mol) resisted MDZ to move toward the water phase, and thus provide driving force for MDZ to precipitate in the IA phase instead of in the water phase. On the other hand, in S-II, the reduction of the interaction energy between SDS and MDZ (Δ*E*_elec_ = –23.84 kJ/mol, Δ*E*_vdW_ = –216.80 kJ/mol) reveals the SDS-MDZ attraction. The reduction in interaction energy between water and MDZ is also detected in S-II. Consequently, the attraction from SDS and water together provided driving forces for the accumulation of MDZ near the interface, and thus assisted the formation of the hollow MDZ-NPs.

**Table 3.** Change of interaction energy (Δ*E*, kJ/mol) between individual components.

Simulation system	Δ*E*	SDS-SDS	SDS-IA	SDS-water	SDS-MDZ	IA-IA	IA-water	IA-MDZ	Water-MDZ	MDZ-MDZ
S-I	Δ*E*_elec_	10.46	11.92	66.48	43.83	868.82	–7302.23	101.19	–33.23	–317.50
	Δ*E*_vdW_	2.66	–1167.43	–6.98	178.93	5157.03	–5281.94	227.20	30.23	–259.04
S-II	Δ*E*_elec_	–1.68	7.31	–320.84	–23.84	410.49	–3848.31	96.43	–92.77	–61.42
	Δ*E*_vdW_	17.26	–476.72	56.43	–216.80	1384.04	–2552.68	409.06	–45.26	–102.24

### Preparation and characterization of MMB4@MDZ nanoparticles

**Figure 2.**
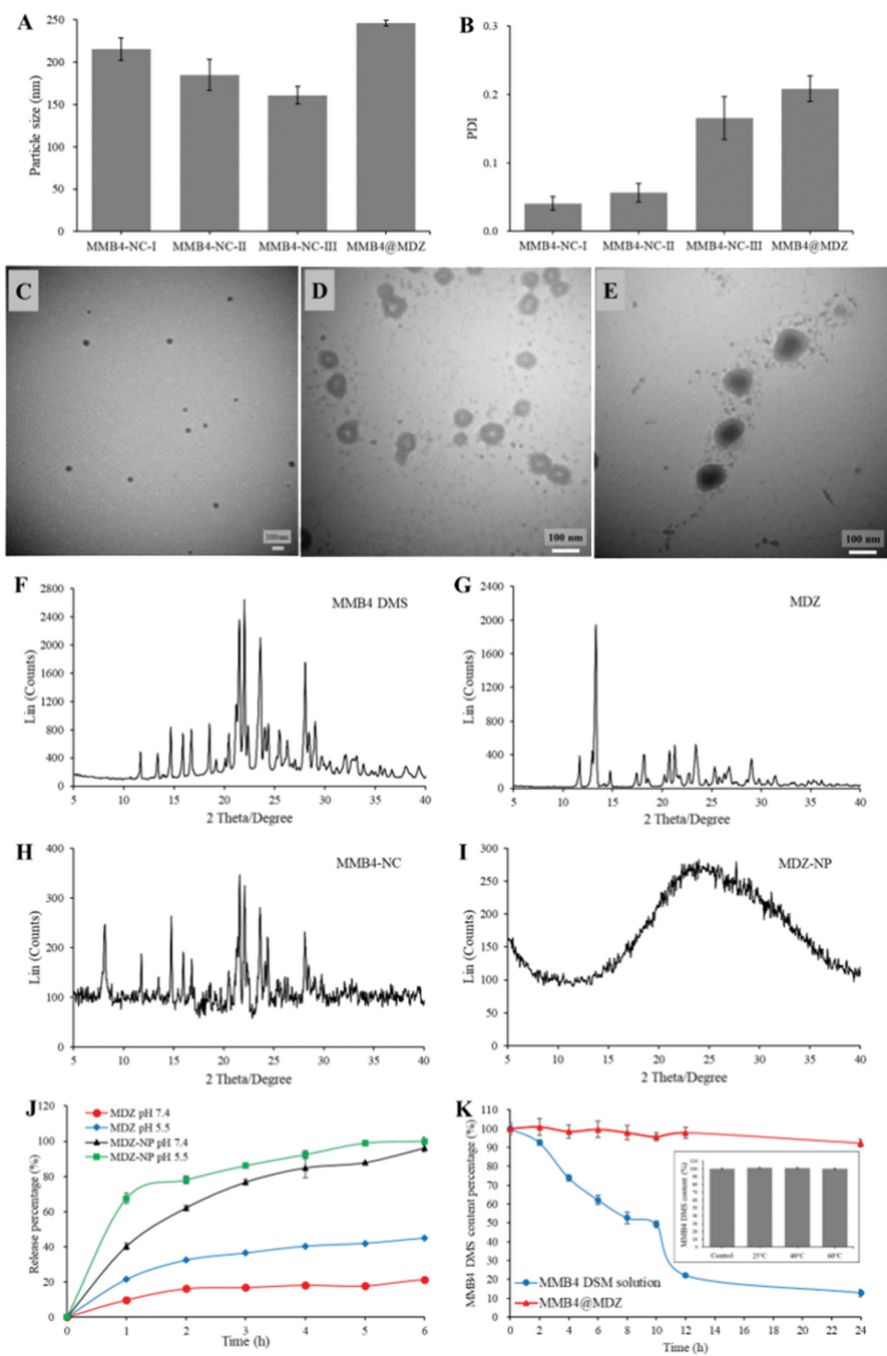
The characterization of MMB4-NCs, MDZ-NP and MMB4@MDZ. DLS measurement (*n* = 3) of (A) particles size and (B) PDI values of MMB4-NC samples and MMB4@MDZ. TEM images of (C) MMB4-NC-III, (D) MDZ-NP, and (E) MMB4@MDZ nanoparticles. XRD pattern of (F) unprocessed MMB4 DMS powder, (G) unprocessed MDZ powder, (H) MMB4-NC-III and (I) MDZ-NPs. (J) Drug releasing profiles of unprocessed MDZ powder and MDZ-NPs. (K) Stability assessment of MMB4 DMS solution and MMB4@MDZ suspension by measuring the content of MMB4 DMS at predetermined time points. The inset shows the result of the stability study of MMB4-NCs suspension (in IA).

MMB4 DMS is chemically unstable in aqueous solution due to hydrolytic cleavage (Dixon et al., 2013). To solve this issue, in this study, MMB4-NCs were prepared in hydrophobic environment to prevent it from degradation. Antisolvent precipitation is a facile but effective approach to produce nanocrystals of poorly water-soluble drugs. In this study, we adopted this technology to produce MMB4-NCs in a hydrophobic organic solvent. In order to explore the optimal synthesis protocol, we have investigated various solvent and antisolvent pairs, and methanol and IA were finally determined to be promising solvent and antisolvent, respectively. MMB4-NCs could be synthesized by simply injecting MMB4 DMS methanol solution into IA without involving stabilizer. MMB4 DMS methanol solutions with different concentrations (0.4, 0.2, 0.1 and 0.05 mg/mL) were used to prepare MMB4-NCs (MMB4-NC-I, MMB4-NC-II, MMB4-NC-III and MMB4-NC-IV). DLS measurement of MMB4-NC-IV resulted a PDI value of 1, indicating a polydisperse size distribution. On the other hand, as shown in Figure 2(A,B), MMB4-NC-I, MMB4-NC-II and MMB4-NC-III all had narrow size distribution and their size decreased with the concentration of MMB4 DMS methanol solution. The TEM image (Figure 2(C)) showed that the smallest MMB4-NC-III had well dispersed spherical morphology with diameter of about 51 nm. Moreover, the XRD pattern of MMB4-NC-III (Figure 2(H)) exhibits the well crystalline structure having same characterization peaks with the unprocessed MMB4 DMS powder (Figure 2(F)). Taking advantages of its small size and narrow PDI, MMB4-NC-III was chosen to be further synthesized into core–shell MMB4@MDZ.

Besides serving as an antisolvent for the preparation of MMB4-NCs, IA is a volatile water-immiscible solvent which is suitable for constructing an O/W emulsion system. According to the simulation results, MDZ molecules tended to assemble near the interface in an IA-based O/W emulsion system. The as-synthesized MDZ-NPs were observed under TEM (Figure 2(D)), and exhibited a hollow sphere-like structure. This is in agreement with the simulation results that the IA droplet served as a soft template and MDZ molecules tended to assemble at near the O/W interfaces. The XRD pattern of MDZ-NPs (Figure 2(I)) appeared to be an amorphous pattern without any characteristic diffraction peak shown in the XRD pattern of the unprocessed MDZ (Figure 2(G)). MDZ is a basic drug due to the nitrogen in the imidazole ring. It has been widely reported that the solubility of MDZ increases considerably in acidic media than in neutral media (Andersin, 1991). Consequently, as shown in Figure 2(J), the unprocessed MDZ powder exhibited faster releasing under pH 5.5 than under pH 7.4. Similarly, the MDZ-NP also released faster under pH 5.5 and nearly 80% were released within 2 h. This quick-dissolution profile should be attributed to the synergic effect of the nanosize and the amorphous nature of MDZ-NP. This acid-accelerated dissolution property is desirable for TfR-mediated delivery across BBB due to the acidic intracellular route. As a result, a TfR-mediated brain-targeting delivery strategy for MMB4 DMS was proposed by utilizing MDZ-NPs. The nanomedicine MMB4@MDZ can be facilely prepared via incorporating MMB4-NCs and the MDZ shell in an O/W emulsion system. The as-obtained MMB4@MDZ had a core–shell structure (Figure 2(E)) with a hydrodynamic size of 245.9 ± 3.1 nm. This MMB4-NC loading strategy can provide a nearly 100% entrapment efficiency (EE), which was significantly higher than previously published drug delivery systems.

Besides the fascinating fast releasing behavior, the eagerly required property of the nanocarrier is the ability to protect MMB4 DMS from degradation. For this purpose, the stability of MMB4-NCs in IA suspension was first studied at different temperatures (25, 40 and 60 °C) to investigate the stability of MMB4 DMS in the O/W emulsion system. Clearly, there was no detectable degradation for all the MMB4-NCs samples. Moreover, the emulsion interface simulation illustrated that the MDZ molecules accumulated in the IA phase (Figure 1(F)), the MMB4-NCs were well preserved in the IA environment during the synthesis process. The MMB4 DMS chemical stability was then evaluated for both MMB4@MDZ suspension and MMB4 DMS aqueous solution under a harsh temperature (60 °C). Due to the protection from MDZ shell, MMB4@MDZ was found to have a fascinating enhancement of the chemical stability of MMB4 DMS with nearly no degradation for up to 12 h, whereas degradation of the dissolved MMB4 DMS in aqueous solution progressed rapidly (Figure 2(K)). In summary, the releasing behavior and stability property made MMB4@MDZ a safe and efficient drug delivery platform. In order to further realize TfR-mediated brain-targeting delivery, CRT was modified onto MMB4@MDZ surfaces to produce CRT-MMB4@MDZ. The coverage of CRT caused a decrease of zeta potential from –39.96 ± 0.71 mV of MMB4@MDZ to –12.05 ± 0.97 mV of CRT-MMB4@MDZ. The modification density of CRT was calculated to be 68.07 mg per mg of MMB4 DMS. As a control sample, Tf-MMB4@MDZ was also prepared with the same method, and the modification density of Tf was calculated to be 301.30 mg per mg of MMB4 DMS.

### Specific binding between CRT-MMB4@MDZ and Tf

**Figure 3.**
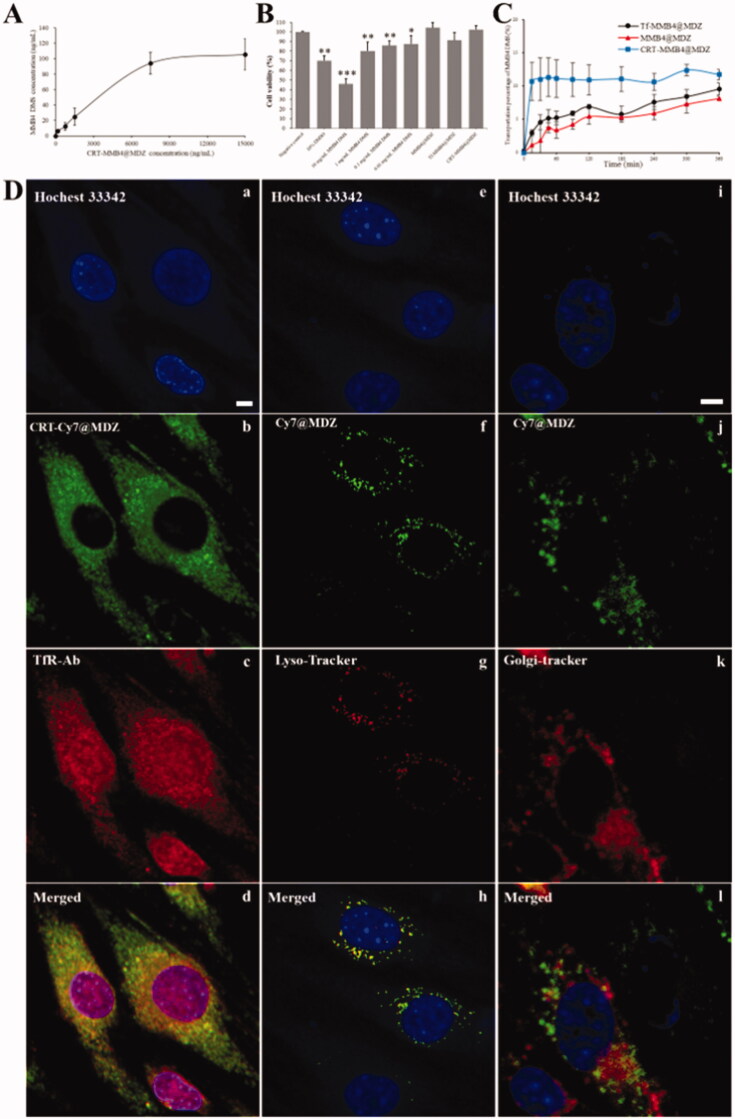
(A) The result of specific binding between CRT-MMB4@MDZ and Tf. (B) MTT results of bEnd.3 cells incubated with MMB4 MDZ solution, MMB4@MDZ, Tf-MMB4@MDZ and CRT-MMB4@MDZ. (C) The transportation ratio profile of MMB4 DMS across the BBB monolayers in different nanoparticle formulations (MMB4@MDZ, Tf-MMB4@MDZ and CRT-MMB4@MDZ). (D) CRT-Cy7@MDZ was added to bEnd.3 cells and co-incubated with TfR-Ab; Cy7@MDZ were added to bEnd.3 cells and co-incubated with Lyso-Tracker and Golgi-Tracker, respectively. The fluorescence in cells was detected by a confocal microscope (scale bar, 5 μm).

The specific binding between CRT-MMB4@MDZ and Tf was demonstrated by an *in vitro* capturing experiment. Briefly, a series of CRT-MMB4@MDZ solutions were applied to a 96-well plate coated with Tf. After incubation and washing, the captured nanoparticles were dissolved in methanol and then the MMB4 DMS concentration was measured. Figure 3(A) plots the resultant MMB4 DMS concentration versus the concentration of applied CRT-MMB4@MDZ solution. It is clear that the resultant MMB4 DMS concentration increased with the concentration of applied CRT-MMB4@MDZ, demonstrating CRT-MMB4@MDZ to be specifically captured by Tf. The control sample BSA-MMB4@MDZ was also investigated in this capturing experiment, but the Tf-coated 96-well plate was not able to capture BSA-MMB4@MDZ nanoparticles. The result of this capturing experiment led to a conclusion that the CRT molecules modified on MMB4@MDZ surfaces enabled CRT-MMB4@MDZ to be able to specifically bind with Tf.

### Cytotoxicity, transepithelial transport, and transcytosis study

To further illuminate the potential of CRT-MMB4@MDZ for TfR-mediated brain-targeting delivery, a series of *in vitro* and *in vivo* experiments were carried out. The cytotoxicity study demonstrated that MMB4 DMS solution had an obvious concentration-dependent cytotoxicity effect which can be greatly reduced by the core–shell nanoparticle strategy (Figure 3(B)). Clearly, MMB4@MDZ, Tf-MMB4@MDZ and CRT-MMB4@MDZ all had negligible cytotoxicity even at a high concentration (1 mg/mL MMB4 DMS-equiv.). These distinct biocompatible nanoparticles were further evaluated for the ability to cross BBB by the transcytosis measurements. The bEnd.3 monolayers were cultured on transwell apparatus, and the successful establishment of an *in vitro* BBB model was verified by high TEER (>200 Ω cm^2^). Figure 3(C) represents the transport ratio profile over a period of 6 h. The permeability of MMB4 DMS solution across the bEnd.3 monolayers was not detectable. In contrast to the solution, the transport of MMB4 DMS was evidently promoted in MMB4@MDZ benefiting from the nanoparticle form. Moreover, Tf-MMB4@MDZ and CRT-MMB4@MDZ both showed increased BBB crossing efficiency and CRT-MMB4@MDZ obviously had excellent performance among all the nanoparticle samples. The TEER values were monitored throughout the study, and the TEER kept higher than 200 Ω cm^2^ throughout the whole experiment procedure, confirming the integrity of the bEnd.3 monolayers. The intracellular localization of CRT-MMB4@MDZ into the bEnd3 cells was surveyed through CLSM microscopy, wherein green and red fluorescence represents CRT-Cy7@MDZ and TfR-Ab, respectively (Figure 3(Db, Dc)). On the merged images, CRT-Cy7@MDZ showed a high colocalization ratio with TfR-Ab (Manders’ coefficient, 0.788). This indicated that the internalization pattern of CRT-Cy7@MDZ was mainly due to TfR mediated endocytosis (Vercauteren et al., 2010). On the other hand, Cy7@MDZ highly colocalized with lysosomes (Manders’ coefficient, 0.609), indicating most of the uptaken Cy7@MDZ were delivered to the lysosomes. In the Golgi group, only a small part of Cy7@MDZ colocalized with Golgi complex (Manders’ coefficient, 0.326). Golgi complex is an important station for cellular secretory function, which involved the transport of entities out of cells. This result suggested that a large portion of Cy7@MDZ tended to be accumulated in lysosome, whereas only a limited amount of Cy7@MDZ could be transported across the bEnd.3 cells.

### *In vivo* imaging

**Figure 4.**
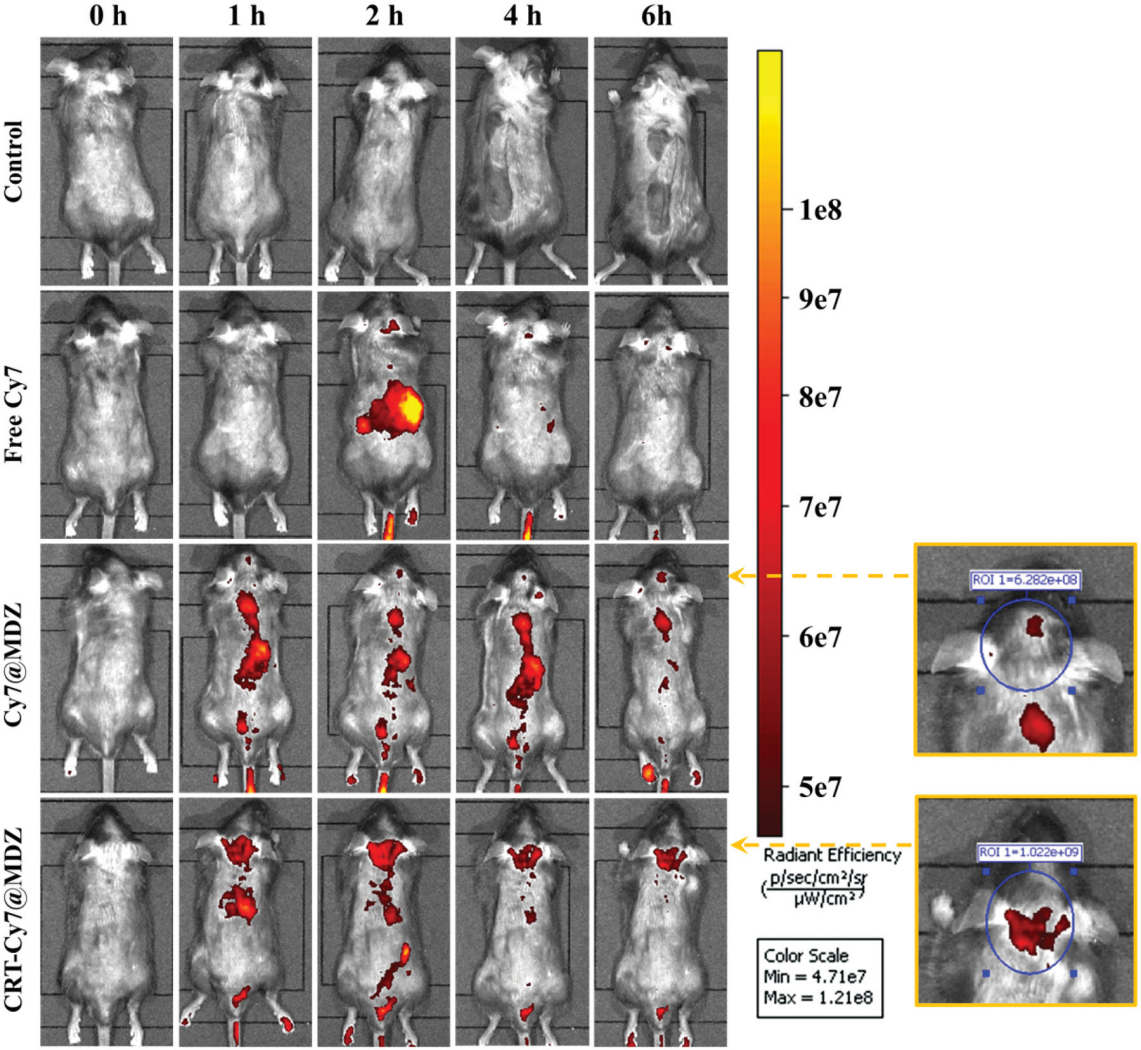
The free Cy7, Cy7@MDZ and CRT-Cy7@MDZ samples were injected to mice via the tail vein, and the *in vivo* near-infrared fluorescent images were detected at different time points.

To evaluate the *in vivo* brain-targeting delivery, a fluorescence imaging system was used to monitor the *in vivo* delivery of Cy7 saline solution, Cy7@MDZ saline suspension and CRT-Cy7@MDZ saline suspension at predetermined intervals (0, 1, 2, 4 and 6 h), with saline set as the control sample (Figure 4). The free Cy7 exhibited distinct accumulation in the liver region at 2 h post-injection, the disappearance of the fluorescent signal after 2 h could be due to the rapid metabolism of free Cy7. On the other hand, Cy7@MDZ and CRT-Cy7@MDZ caused accumulated fluorescent signal in the brain region. It also revealed that CRT-Cy7@MDZ accumulated in brain more superior to Cy7@MDZ. ROI analysis was adopted to quantify the fluorescence intensity of brain region at 6 h post-injection, the fluorescence of CRT-Cy7@MDZ treated mice was 1.6-fold more than that of the Cy7@MDZ, which was consistent with the *in vitro* transport study results.

### *In vivo* pharmacokinetics and biodistribution

**Figure 5.**
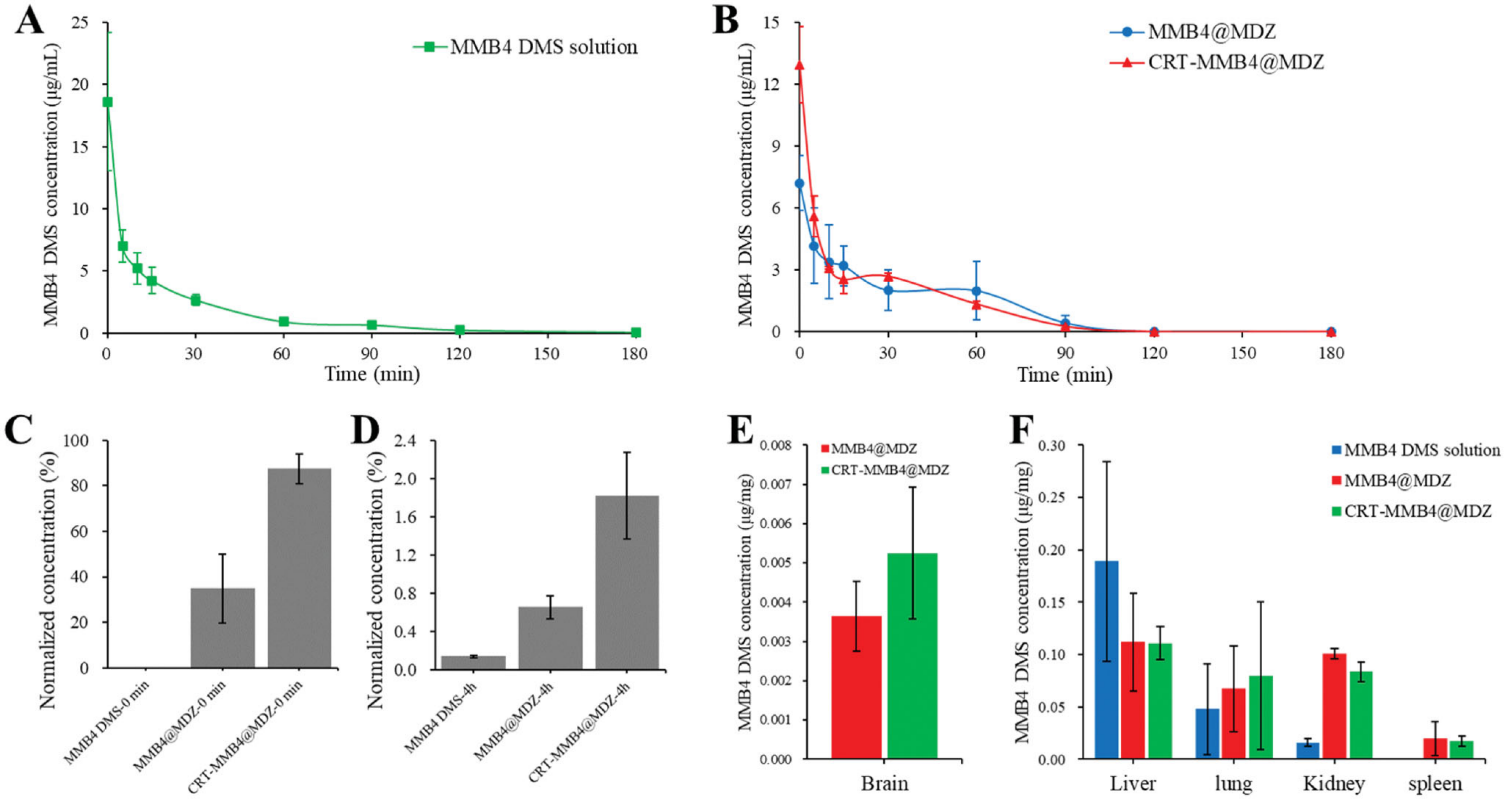
*In vivo* pharmacokinetics profiles of (A) MMB4 DMS solution, and (B) MMB@MDZ and CRT-MMB@MDZ. The normalized MMB4 DMS concentration in CSF (C) at 0 min post-injection and (D) at 4 h post-injection. Tissue distribution of the free MMB4 DMS and the MMB4 DMS delivered via MMB@MDZ and CRT-MMB@MDZ (E) in brain and (F) in other organs. Data are presented as the average ± standard error (*n* = 3).

The pharmacokinetic study was performed by analyzing the MMB4 DMS concentration in the blood circulation of rats at specific time durations via i.v. injection of MMB4 DMS solution, MMB4@MDZ and CRT-MMB4@MDZ into SD rats (Figure 5(A,B)). In all the three groups, MMB4 DMS achieved complete plasma clearance after 3 h of injection. To further assess the ability of CRT-MMB4@MDZ to cross the BBB *in vivo*, the MMB4 DMS concentration in CSF was measured. Figure 5(C,D) plots the CSF MMB4 DMS concentration normalized to the 0 min blood concentration of MMB4 DMS. First, the MMB4 DMS concentration in CSF was measured right after injection (Figure 5(C)). The hydrophilic free MMB4 DMS was difficult to cross BBB, and thus, exhibited no detectable MMB4 DMS in CSF. On the contrary, the nanoparticles (MMB4@MDZ and CRT-MMB4@MDZ) successfully transported across BBB and resulted in quite high MMB4 DMS concentration in CSF. What is more, CRT-MMB4@MDZ (normalized MMB4 DMS concentration in CSF, 87.40 ± 6.42%) performed overwhelming superiority than MMB4@MDZ (normalized MMB4 DMS concentration in CSF, 34.85 ± 15.21%). Subsequently, the MMB4 DMS concentration in CSF was measured again after MMB4 DMS was completely cleared from blood (4 h after injection). After four hours of delivery, a small amount of free MMB4 DMS was delivered across BBB resulting in 0.14 ± 0.013% normalized MMB4 DMS concentration in CSF (Figure 5(D)). As to the MMB4@MDZ and CRT-MMB4@MDZ group, the normalized MMB4 DMS concentration in CSF decreased to 0.65 ± 0.12% and 1.82 ± 0.45%. The biodistribution of MMB4 DMS was further quantified at 4 h post-injection. As shown in Figure 5(E), both MMB4@MDZ and CRT-MMB4@MDZ had obvious accumulation in brain, and CRT-MMB4@MDZ exhibited about 1.5-fold higher brain drug concentration than MMB4@MDZ. This explained the decrease of their MMB4 DMS level in CSF at 4 h post-injection. On the other hand, free MMB4 DMS mainly accumulated in liver, lung, and kidney, and had no detectable accumulation in brain. In summary, the CRT-MMB4@MDZ injection resulted in higher MMB4 DMS level in CSF and higher brain drug concentration, indicating the distinct brain-targeting delivery effect of CRT-MMB4@MDZ.

### Analysis of protein corona

Generally, nanoparticles formed protein corona in blood owing to the high surface free energy, and it is the protein corona that crucially determined the *in vivo* fate of nanoparticles (Monopoli et al., 2011). In recent years, protein corona targeting was proposed for nanoparticle delivery, where the surface-modified nanoparticles can specifically bind with special endogenous protein in the bloodstream to realize protein corona-mediated targeting delivery (Yousefpour et al., 2018; Huo et al., 2020). As an iron-mimic cyclic peptide, CRT is able to selectively interact with Tf through a non-canonical allosteric binding mechanism (Staquicini et al., 2011). A comprehensive characterization of the plasma protein corona was performed to compare MMB4@MDZ and CRT-MMB4@MDZ. After 2 h incubation in plasma, a protocol combining membrane ultrafiltration and high-speed centrifugation was used to isolate the PC-NP complexes. A comprehensive analysis of the protein corona composition was carried out by LC–MS/MS and the 20 most abundant proteins were presented in Table 4. Surprisingly, MMB4@MDZ evolved into a protein corona with Tf being the seventh most-abundant corona protein. This explained the *in vivo* brain-targeting effect of MMB4@MDZ. On the other hand, Tf is the eighth most-abundant corona protein for CRT-MMB4@MDZ. Protein coronas of nanoparticles have been widely reported to be complex and variable, and Tf was not inevitably included into the top 20 most abundant corona proteins (Aggarwal et al., 2009; Cai & Chen, 2019; Richtering et al., 2020). In this context, CRT-MMB4@MDZ and MMB4@MDZ formed protein coronas with more abundant Tf relative to other types of nanoparticles. Protein corona architecture is a multilayer assembly of proteins that undergo dynamic exchanges with free proteins *in vivo* (Chen et al., 2017; Zhang et al., 2020). Consequently, Tf in the protein corona of CRT-MMB4@MDZ suffered association with other types of proteins and frequent exchanges with serum proteins. This might be the reason that Tf was not, as expected, predominantly abundant in the corona of CRT-MMB4@MDZ. Although Tf in the protein corona of CRT-MMB4@MDZ is not superior than that in the protein corona of MMB4@MDZ, CRT-MMB4@MDZ exhibited better brain-targeting effect. In recent years, researches about the spatial organization of protein coronas have come out and the randomly oriented corona proteins have been reported (Kelly et al., 2015; Liessi et al., 2021). It is reasonable to speculate that the Tf molecules bind on MMB4@MDZ with random orientation, resulted in only part of the Tf molecules orienting their TfR-binding sites to the outer-surface. On the other hand, CRT on MMB4@MDZ surfaces specifically bound with Tf and leads the TfR-binding site of Tf present on protein corona surface. In summary, taking advantages of the well oriented Tf in the protein corona, CRT-MMB4@MDZ performed a satisfactory efficiency in brain delivery.

**Table 4.** The 20 most abundant proteins identified in the protein corona of MMB4@MDZ and CRT-MMB4@MDZ.

No.	MMB4@MDZ		CRT-MMB4@MDZ	
	Proteins in protein corona	Composition of corona (%)	Proteins in protein corona	Composition of corona (%)
1	Fibrinogen beta chain	10.40	Alpha-1-macroglobulin	26.78
2	Fibrinogen gamma chain	8.46	Fibrinogen beta chain	8.05
3	Serum albumin	6.70	Fibrinogen gamma chain	5.91
4	Albumin	6.52	Complement C3	5.46
5	Ac1873	6.41	Ac1873	3.95
6	Ig gamma-2B chain C region	4.99	Alpha-1-inhibitor 3	3.66
7	Serotransferrin	2.67	Apolipoprotein A-I	3.16
8	Ig lambda-2 chain C region	1.82	Serotransferrin	2.82
9	Alpha-2-HS-glycoprotein	1.79	Ig gamma-2B chain C region	2.33
10	Fibronectin	1.78	Apolipoprotein E	2.15
11	Vitronectin	1.41	Adiponectin a	1.69
12	Anionic trypsin-1	1.35	Histidine-rich glycoprotein	1.61
13	Alpha-1-macroglobulin	1.26	Vitronectin	1.50
14	Apolipoprotein E	1.15	Globin c2	1.22
15	Apolipoprotein A-I	1.01	Apolipoprotein A-IV	1.19
16	Hemopexin	0.99	Ig lambda-2 chain C region	1.02
17	Alpha-1-inhibitor 3	0.97	Inter alpha-trypsin inhibitor	0.92
18	Alpha-1-antiproteinase	0.87	CD5 antigen-like	0.87
19	Apolipoprotein C-III	0.85	Hemopexin	0.78
20	Haptoglobin	0.83	Serum albumin	0.77

Overall, the results above altogether suggested that the MMB4@MDZ nanoparticles could enhance the chemical stability of MMB4 DMS and CRT-MMB4@MDZ performed preferential brain-targeting effect. This targeting effect could be attributed to the CRT modified nanoparticle surface, which tends to adsorb the endogenous Tf in blood to form a Tf-abundant corona, and thus achieve brain-targeting delivery via TfR. Compared to other previous works on brain-targeting delivery of AChE reactivator, CRT-MMB4@MDZ nanoparticles had significant advantages in simplifying preparation procedure, excellent drug loading and reducing potential side effects of nonactive excipients. Based on that, we considered that the CRT-MMB4@MDZ nanomedicine have significant prospects in bench-to-bedside translation.

## Conclusions

In this study, the water soluble MMB4 DMS was facilely synthesized into spherical MMB4-NCs with high crystallinity and uniformed size by adopting IA as antisolvent in the antisolvent precipitation method. IA also served as an organic phase in an emulsion-solvent evaporation technology for preparation of MDZ-NPs. The as-prepared MDZ-NPs were shown to have a hollow structure, and the molecular mechanism driving the formation of this hollow structure was proposed by adopting molecular simulation. The versatile IA provided a chance to synthesize the core–shell nanoparticle MMB4@MDZ through combining the antisolvent precipitation method and the emulsion-solvent evaporation method. The core–shell MMB4@MDZ endowed with excellent stability of MMB4 DMS and appropriate nanoscale size was considered as a brand-new carrier-free nanomedicine, which was then modified with an iron mimic peptide CRT to produce CRT-MMB4@MDZ. Coupled with the Tf binding effect of CRT, the CRT-MMB4@MDZ can obtain a Tf-abundant protein corona in plasma. Owing to the Tf-abundant corona, this CRT-MMB4@MDZ nanomedicine could cross BBB via TfR, contributing a brain-targeting drug delivery. This work provided endogenous protein corona-mediated targeting strategy for the core–shell nanomedicine to realize superior biosafety profile and brain-targeting delivery of MMB4 DMS. While herein we report a combinatorial protocol to assemble a water-soluble core and a hydrophobic shell, the potential of this strategy to fabricate multilayered nanomedicine for controlled sequential drug delivery cannot be ruled out.
